# Bacterial Flagella: Twist and Stick, or Dodge across the Kingdoms

**DOI:** 10.1371/journal.ppat.1004483

**Published:** 2015-01-15

**Authors:** Yannick Rossez, Eliza B. Wolfson, Ashleigh Holmes, David L. Gally, Nicola J. Holden

**Affiliations:** 1 Cellular and Molecular Sciences, James Hutton Institute, Dundee, United Kingdom; 2 Division of Infection and Immunity, The Roslin Institute, University of Edinburgh, Easter Bush, United Kingdom; Stony Brook University, UNITED STATES

## Abstract

The flagellum organelle is an intricate multiprotein assembly best known for its rotational propulsion of bacteria. However, recent studies have expanded our knowledge of other functions in pathogenic contexts, particularly adherence and immune modulation, e.g., for *Salmonella enterica, Campylobacter jejuni, Pseudomonas aeruginosa*, and *Escherichia coli*. Flagella-mediated adherence is important in host colonisation for several plant and animal pathogens, but the specific interactions that promote flagella binding to such diverse host tissues has remained elusive. Recent work has shown that the organelles act like probes that find favourable surface topologies to initiate binding. An emerging theme is that more general properties, such as ionic charge of repetitive binding epitopes and rotational force, allow interactions with plasma membrane components. At the same time, flagellin monomers are important inducers of plant and animal innate immunity: variation in their recognition impacts the course and outcome of infections in hosts from both kingdoms. Bacteria have evolved different strategies to evade or even promote this specific recognition, with some important differences shown for phytopathogens. These studies have provided a wider appreciation of the functions of bacterial flagella in the context of both plant and animal reservoirs.

## Introduction

The prokaryotic flagellum is best known as a motility organelle responsible for bacterial movement and necessary for chemotaxis [1]. An extraordinary multisubunit organelle, complex in its regulation and assembly, the flagellum has been the subject of extensive research over the past four decades and a central topic of evolutionary debate [2]. Ongoing research is still revealing surprises in various aspects, from assembly to function [3,4].

A switch in lifecycle from sessile to motile, e.g., exiting biofilms or established microcolonies in response to depletion in nutrients, effectively deems individual flagellate cells as pioneers, in search of more favourable environments. Often this means exploration of new hosts, habitats or niches. In this respect, flagella can be considered an early stage colonisation factor. Flagella from a variety of bacteria have been shown to bind to a diverse array of animal and plant substrates, and this review focuses on recent advances in our understanding of how flagella impact host-microbe interactions. The flagellum filament, attached to a transmembrane motor complex, is a long helical structure made up of hundreds of subunits of the flagellin protein, encoded by *fliC* (or homologues). The copy number and location on the bacterial cell surface varies between species. Here, adherence is described for intact flagella, while aspects of recognition and evasion relate to the flagellin protein. Polymorphisms in flagellin has provided a mechanism of sub-species differentiation, based on the “H” (Hauch) antigen type.

## Twist and Stick: Interactions with Host Tissues

A role for flagella-mediated adherence has been demonstrated in many different plant species and animal infection models, for both pathogenic and opportunistic bacteria [4,5]. These results reveal a significant role for flagella during colonisation and, consequently, environmental transmission. Other factors also facilitate adherence, including electrostatic charge, or specific fimbrial-mediated interactions that may occur at subsequent stages and confer tissue tropism.

Initial flagella interactions drive formation of bacterial biofilms or microcolony communities and maintain their structure along colony surfaces through physical interactions [6]. Additionally, and perhaps surprisingly, these organelles can act as probes of uneven surfaces during biofilm formation, attaching within crevices [7]. Recent work demonstrated that individual cells, and consequently the growing colony, effectively become tethered to artificial surfaces that resemble microvilli; a scenario that is very likely to hold true for interactions with true biotic surfaces [7].

**Figure 1:**
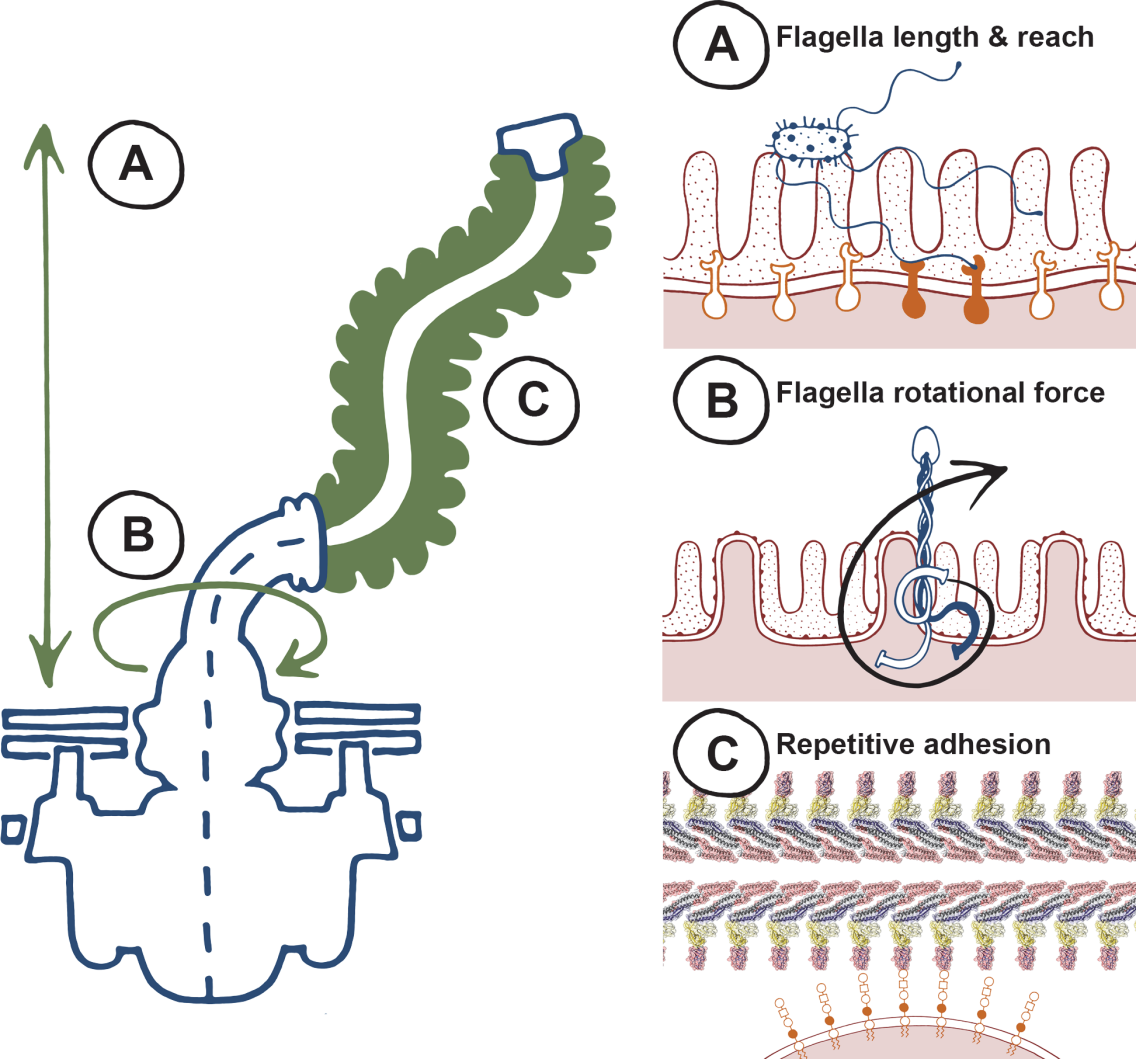
The biophysical properties of flagella, to “twist and stick,” lend themselves towards nonspecific adhesion. Left, a summary of key characteristics of the flagella apparatus that are advantageous for adherence, and right, specific properties highlighted on the left. (A) Flagellum length results in a long reach towards colonization surfaces as an early-stage anchor—higher affinity binding can occur at closer proximity with specific adhesins and receptors. (B) Flagella rotation generates force that promotes membrane interactions during initial adherence. (C) Flagella are highly repetitive structures, non-specific low affinity binding can result in adhesion at high avidities.

There is a large body of published work for flagella-mediated adherence, yet there are very few examples of specific interactions where both flagella and host determinants have been formally dissected. Instead, there is an emerging theme of nonspecific interactions, which has been challenging to investigate and likely relates to the biophysical properties of flagella (Fig. 1). Firstly, flagella organelles are long filaments that can reach up to 20 μm from the bacterial cell surface (Fig. 1A). It is therefore logical that flagella can be exploited as adhesive scaffolds and are involved in initial probing of surfaces as an early colonisation factor. Secondly, its motor can spin flagella filaments at speeds in excess of 15,000 rpm (Fig. 1B), which not only increases the chances of the filament coming into contact with surfaces, but also ensures it does so with force [8]. This is consistent with evidence that some flagella aren’t adhesive, but are involved in cellular binding and invasion in processes distinct from providing niche proximity through propulsion. In the absence of specific protein receptors, observation of intercalation and penetration into plant and animal membrane lipid layers by flagella could also be explained by this phenomenon. Thirdly, the flagellum filament is a polymeric structure, comprised of repeating epitopes of one or more flagellin types (Fig. 1C). Repeating epitopes are high avidity by definition: low affinity ionic interactions can be consolidated, amplified, and relevant if the binding substrate is also repetitive. With very few exceptions (innate immune receptors being the most notable), published examples of “specific” flagella binding interactions are with factors that are repetitive, such as polymeric proteins, proteoglycans, glycolipids, and phospholipids. Flagella therefore appear to be a tool with general properties that can be adapted to pathogenic colonisation of a diverse range of niches across plant and animal kingdoms.

### Adhesion to plant tissues

Flagella-mediated adherence to plant tissues has previously been described for phytobacteria, such as *Pseudomonas syringae* to bean seedlings [9] and nitrogen-fixing bacteria *Azospirillum brasilense* to wheat roots [10]. Fresh vegetables or fruits are now recognised as secondary hosts involved in the transmission of human pathogenic bacteria through the food chain [11,12]. Flagella play a role in adherence to plants for multiple human pathogens, including *Salmonella enterica*, pathogenic *Escherichia coli* and *Listeria monocytogenes*. Attachment of *S. enterica* to basil leaf epidermis appears to be serovar specific, such that flagella mutants of serovar Senftenberg were significantly reduced in attachment compared to an isogenic parent, whereas *S. enterica* serovar Typhimurium (*S*. Typhimurium) mutants were not, presumably because of other adherence factors [13]. *L. monocytogenes* flagella adhere to sprouted seeds of alfalfa, broccoli, and radish [14]. Interestingly, a motility deficient mutant (*motAB*) was not deleteriously affected in adherence compared to the parental strain, demonstrating that flagella-mediated binding did not require chemotaxis or flagella rotation. Chemotaxis towards and tropism for stomatal guard cells has been reported for *S*. Typhimurium on lettuce leaves [15] and enteroaggregative *E. coli* (EAEC) on rocket leaves, respectively, although mutation of EAEC *fliC* did not reduced adherence to epidermal leaf cells [13]. Flagella expressed by enterohaemorrhagi*c E. coli* (EHEC) O157:H7 and enterotoxigenic *E. coli* (ETEC) mediate attachment to rocket, spinach, lettuce, and basil leaves [16,17]. The requirement of flagella for systemic spread of *S. enterica* or EHEC from root to shoot of *Arabidopsis thaliana* plants is likely to relate more to motility and dissemination rather than to adhesion [18].

### Adhesion to mammalian tissues

The role of flagella in bacterial adherence to mammalian hosts has been demonstrated for various bacterial species in a number of hosts [5]. Dissection of the different contributions of flagella for motility, adherence, and invasion is complex. This is exemplified by studies with *Pseudomonas aeruginosa*, where flagella-dependent uptake by macrophages occurs, despite only a marginal role for flagella-mediated binding to the cells [19]. In motile *L. monocytogenes*, flagella were required for optimal adherence and invasion of CaCo-2 cells, even after centrifugation onto monolayers, although the flagella alone were not adhesive [20]. This suggests that in this case rotational force was necessary for adherence and invasion, distinct from bacterial propulsion.

Flagella binding can be host tissue-specific for particular flagellar serotypes. Flagella from attaching and effacing bacteria EHEC O157:H7 and enteropathogenic *E. coli* (EPEC) O127:H6 have both been shown to be involved in the adherence to porcine gastric mucins and bovine primary intestinal epithelial cells and explants [21,22]. However, H7 serotype flagella appear to have a particular tropism for rectal epithelial cells. Complementation of an *E. coli* O157:H7 *fliC* mutant with *fliC*_H7_ restored adherence in this tissue to wild-type levels, whereas no significant effect was seen for the mutant complemented with an alternative *fliC* type (H6). An earlier study reported that while purified H2 and H6 flagella were adhesive to HeLa cells, H7 flagella was not [23], illustrating the importance of using relevant cell lines for infection models.

For some species, secretion of nonflagellin proteins occurs via the flagella apparatus, similar to that of the nonflagellar T3SS. For example, flagella-mediated secretion has been demonstrated for *Campylobacter jejuni* FlaC, an adhesin that facilitates binding to Hep-2 cells [24].

### Interaction with mucus

Epithelial mucosal tissues act as a barrier between the animal host and its environment. Epithelial barrier cells are often coated by a layer of high viscosity mucus under a low viscosity mucus layer, both composed of water and a plethora of defensive compounds such as mucins. Mucins are highly glycosylated glycoproteins mainly composed of *O*-linked oligosaccharides, acidic monosaccharides with sialic acid, or modifications of other components, such as sulphate groups.

Flagellar-mediated motility through (animal) mucus can be a prerequisite for successful mucosal colonisation [25]. *Helicobacter pylori* swims through the mucus barrier, burrowing deep in the gastric mucus layer to escape the acidity of the stomach [26–28]. Yet, although *Helicobacter* flagella may be required for colonisation, there is no evidence of specific attachment of flagella to epithelial cells [29]. Indeed, mucus can repress motility, as with EHEC, ETEC, and *S*. Typhimurium, where incubation with mucus results in a down-regulation of flagella-associated genes or reduced swarming motility [30–32]. For *Vibrio cholerae*, passage through mucus can trap flagella, causing them to break and release the anti σ^28^-factor FlgM. This derepresses *fliA*, which encodes σ^28^, allowing optimal expression of late-class flagella genes, perhaps to replace the broken flagellum. Additionally, σ^28^ represses HapR, part of a quorum sensing network in *V. cholera*, allowing expression of genes required for toxin production [33]. This is an elegant mechanism of sequential and appropriate expression that occurs where expression of a single flagellum is needed and linked to discrete points of the infection cycle.

There are various reports of interactions between flagella and mucus. The opportunistic pathogen *Stenotrophomonas maltophilia* has been described to bind mouse tracheal mucus via its flagella [34]. Similarly, H6 and H7 flagellins from EPEC and EHEC, respectively, adhere to bovine mucus [35]. A certain FliD cap protein type (B-type) of *P. aeruginosa* strains binds to respiratory-derived mucins via the Lewis X determinants [36,37]. Probiotic *E. coli* strain Nissle flagella adhere to human intestinal mucus through gluconate [38], although gluconate is not directly associated with gastric nor intestinal human mucins [39,40]. It is possible that resident microbial communities degrade complex host mucus glycans and release gluconate into the mucus layer, providing a gluconate “decoy” as a means of competition [41,42]. In contrast, there are relatively few reports showing direct interactions of bacterial components with plant mucilage [43,44] and none as yet for flagella, although one could conceive the potential for such interactions. Mucilage secreted by plant root tip cells, to facilitate expansion of the growing root and provide protection against many microbes, is functionally but not structurally analogous to mucus [45].

### Molecular targeting

While there are many studies demonstrating a role for flagella in adherence and colonisation of tissues, few have identified or characterised specific targets used by the flagella for binding. In animal cell models, *P. aeruginosa* flagella can recognise the basolateral surface of polarized lung epithelial cells through heparan sulphate, a highly sulphated proteoglycan [46]. A recent study supports a new role for surfactant protein A in binding and enhancing the clearance of *P. aeruginosa* flagellin, mediated in part by enhanced IL-1β production [47]. Furthermore, the *P. aeruginosa* flagellum has been described to bind glycolipids monosialoganglioside (GM) or disialoganglioside (GD), playing a role in the pulmonary infection where flagellin binding to GM1 was greater than to asialoGM1 [48]. In contrast, flagella from *E. coli* O113:H21 did not contribute to adherence, but did contribute to invasion via asialoGM1 [49].

Flagella-mediated adherence does not have to be direct; flagella can act as extended molecular scaffolds for other secreted adhesins. EtpA, a two-partner secretion exoprotein adhesin from ETEC, interacts with the conserved region of flagellin, by accessing the uncapped flagellum tips. This “bridge” allows the indirect adhesion of the flagella to intestinal mucosal tissues [50].

Recent work has highlighted the importance of membrane interactions in flagella-mediated adherence to plant cells. H6, H7, and H48 flagellins from *E. coli* were shown to bind algae sulphated polysaccharides and membrane phospholipids in an ionic charge-dependent manner. Specificity for phospholipid recognition allows flagella to intercalate into plant plasma membranes [51], in a manner that may be similar to the flexible, elongated type-III secretion system of phytopathogenic bacteria. The underlying mechanism is likely to be conserved with other eukaryotic hosts and thus a generic method of host–microbe interaction. As demonstrated with *L. monocytogenes* [20], the rotational force of the flagella may promote interactions and aid penetration of plant cell walls. Flagella rotation is also important for *S*. Typhimurium interactions with HeLa cells in a process called “near-surface swimming” [52].

## Dodge: Immune Recognition

Flagellar-mediated host interactions incur a cost, as conserved regions in flagellin monomers are potent inducers of innate immune responses in vivo, across kingdoms. Consequently, recognition of flagellin leads to the greater clearance of flagellated versus nonflagellated strains [20,53–55]. Therefore, there are many immunomodulatory strategies, the simplest being alteration of flagella production and selection of bacteria that are less flagellate upon host-cell contact [56].

### Structural recognition

**Figure 2:**
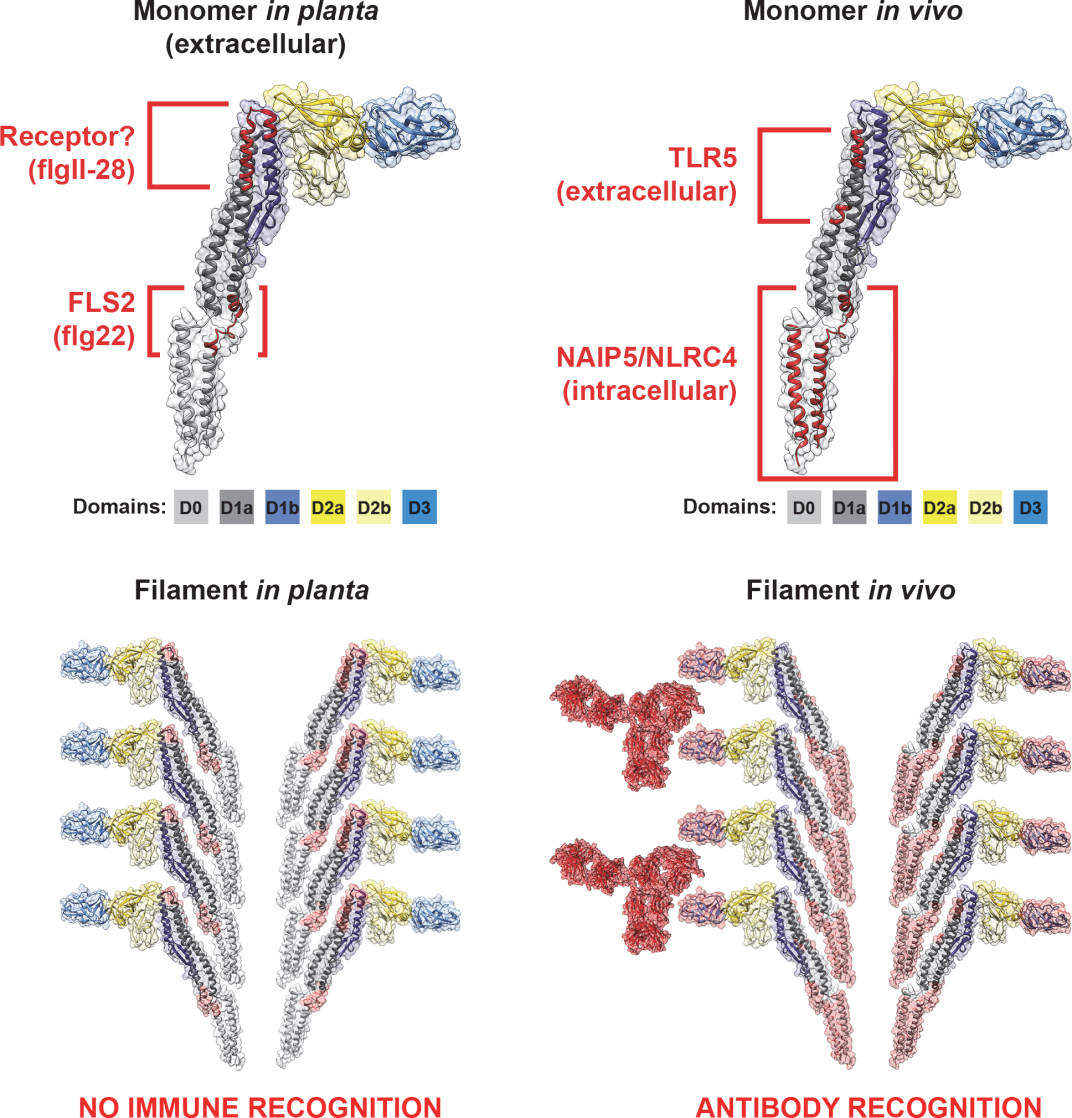
Cross-kingdom immune recognition of flagellin structures. Top: backbone of the key residues of flagellin recognized by plant (left) and animal (right) innate immune receptors are highlighted in red. FliC from *S. enterica* is presented as a “model” flagellin, with reports for recognition by both TLR5 and FLS2 receptors. These residues are superimposed on the solved flagellin structure (PDB# 1UCU) in UCSF Chimera [113]. Surfaces and backbone are coloured according to previously assigned structural domains as indicated below each monomer [57]. Bottom: recognition of flagella filaments by plant (left) and animal (right) innate immune receptors does not occur as key residues (surfaces highlighted in red) are hidden within the filament structure. However, immune recognition still occurs in animals via antibody recognition of the D3 domain.

The flagellin monomer is organised into four connected domains designated D0, D1, D2, and D3 (Fig. 2) [57]. To form these domains, flagellin peptides fold back on themselves, like an elaborate hairpin, with the termini associated with one another. The D2–D3 domains are highly variable and generate the antigenic diversity described as H-serotypes [58–60]. The termini that form the D0–D1 domains are well conserved across all bacterial flagellins. Both N- and C-termini are rich in hydrophobic residues, which form coiled–coil interfaces that allow them to associate with one another; these interfaces are also required for filament polymerisation [3,61,62]. Importantly, the flagellin needs to be disassociated to make D0–D1 accessible to innate immune receptors, in contrast to the polymeric structure involved in binding interactions. Flagellin recognition occurs in plant cells [63] and animal cells of both invertebrates [64] and vertebrates [65]. The receptors for these conserved regions are Toll-like receptor 5 (TLR5) for extracellular flagellin [66–68] and NAIP5-NLRC4 for intracellular flagellin [69] in vivo, and Flagellin sensitive 2 (FLS2) receptor for flagellin in planta (Fig. 2) [70].

Plant and mammal cell membrane receptors share the same architecture of extracellular leucine-rich repeats (LRR) and cytoplasmic association with a conserved family of serine-threonine kinases, although the LRR domains of those two receptors are very divergent [71]. Immune recognition of flagellin is based on highly conserved motifs that span diverse bacterial species (Fig. 2). TLR5 interacts with two short flagellin peptides from both termini (LQRIRELAVQ and LGAIQN) [67,72]. The D1 domain makes a substantial contribution to both high-affinity binding and TLR5 signalling, whereas D0 contributes to TLR5 signalling, but has little or no effect on binding [73]. In plants, FLS2 recognises a conserved N-terminal 22 amino acid peptide in the D0 domain (QRLSTGSRINSAKDDAAGLQIA), termed flg22, derived from *P. syringae* pv. *tabaci* [74,75]. A newly described flagellin-derived MAMP, flgII-28 (located in the D1 domain, Fig. 2), has been shown to be recognized independently of FLS2 by tomato and other solanaceous plants, but not by *Arabidopsis* [76,77].

Flagellin can also be recognised intracellularly in animal cells through another pathway involving receptors from the NOD-like receptor (NLR) family of intracellular pattern recognition or signalling molecules. The NLR apoptosis-inhibitory protein-5 (NAIP5), binds to 35 amino acids from the C-terminus and 52 amino acids from the N-terminus conserved regions of flagellin [78,79]. Unlike mammalian cells, plant cells do not have a cytoplasmic receptor to detect flagellin, as demonstrated by artificial delivery of phytopathogenic bacterial flagellin into the cytosol [80]. This feature may have evolved to prevent exploitation by necrotrophic pathogens that gain from death-associated immune responses.

### Immune evasion by flagella

**Figure 3:**
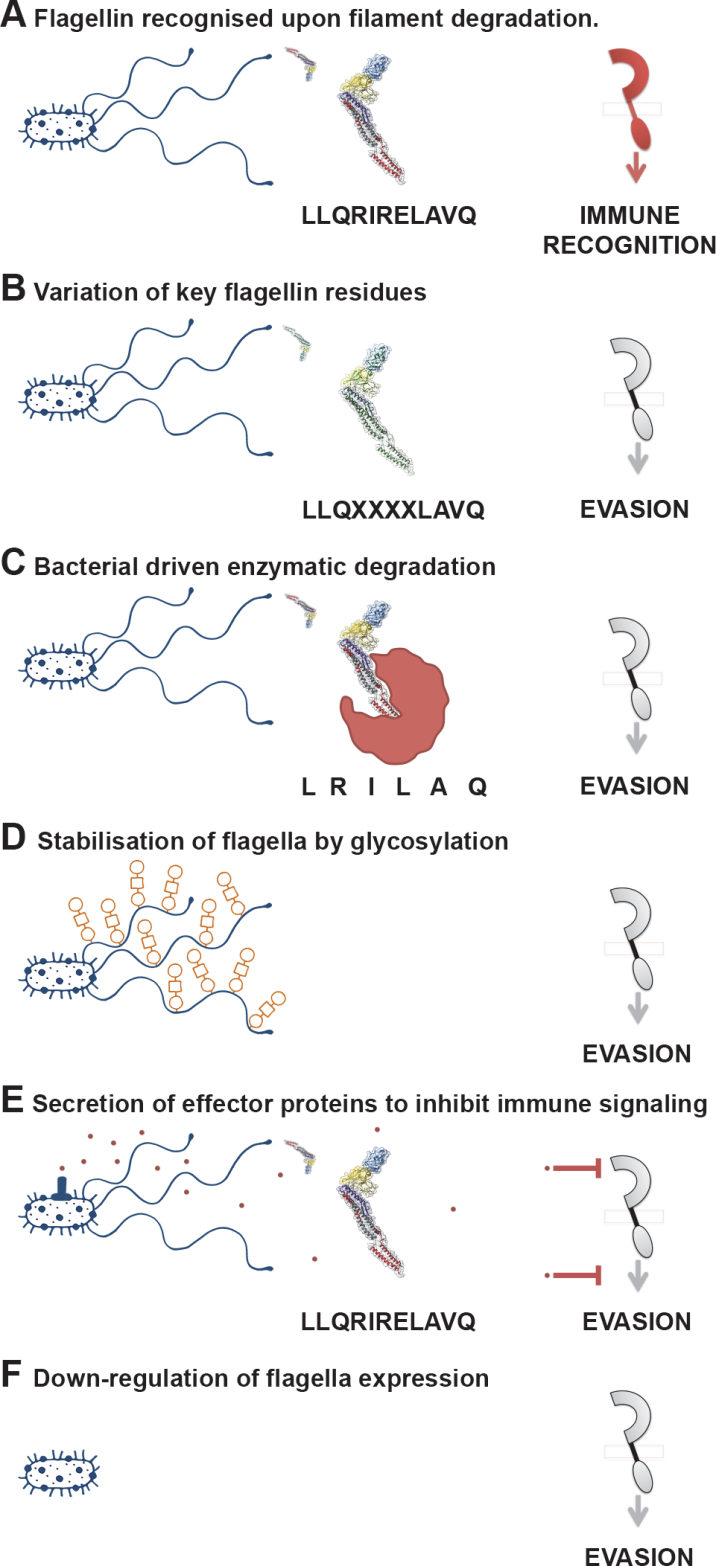
A variety of mechanisms employed to “dodge” the flagellin innate immune response. (A) Flagella filaments degrade, releasing monomeric flagellin, the residues of which are recognised by receptor TLR5, NLRC4, or FLS2, resulting in cytokine release or PTI. The example residues and receptor (right) shown are involved in TLR5 recognition. (B) Flagellin recognition by TLR5, NLRC4, or FLS2 is evaded by variation in key residues involved in flagellin detection, which can necessitate compensatory mutations. (C) Bacteria secrete enzymes that specifically target and degrade monomeric flagellin, preventing its recognition by TLR5, NLRC4, or FLS2. (D) Post-translational glyosylation of flagellin is thought to enhance flagella stability; reduced release of flagellin from flagella filaments will result in reduced recognition by TLR5 or FLS2. (E) Bacteria secrete effector proteins that interfere with TLR5, NLRC4, or FLS2 recognition either by direct inhibition of receptor expression or binding, or by inhibition of downstream signalling pathways. (F) Bacteria down-regulate or switch off flagella expression when motility and/or binding are no longer required.

Since flagellin is such an important immunogen, bacteria have evolved multiple strategies to avoid or evade recognition (Fig. 3). Some pathogens enocode multiple flagellin types, which may relate to evasion or even niche versatility [81]. The majority of *S. enterica* encode two flagellin types (phase 1 & 2), under phase variable control of expression [81–84]. A third flagellin gene, *flpA*, has also been described for a particular *S. enterica* isolate [84]. However, control of expression may be of greater importance in evasion. Nonflagellate mutants out-competed flagellated EHEC O157:H7 colonisation in cattle [85], and nonflagellated strains of plant pathogens *Xanthomonas fuscans* subsp. *fuscans* are isolated from natural epidemics of plant disease [86]. Furthermore, attenuation of infection occurred when phase 2 flagella were constitutively expressed in *S*. Tyhimurium, following oral or intravenous inoculation of mice were in the mouse model of infection [87].

Amino acid substitution enables several animal bacterial pathogens and commensals to reduce activation of TLR5 signalling [88]. Differences in recognition and subsequent signalling elicited by different flagellins suggests host discrimination between pathogenic and commensal bacteria, as a nonpathogenic strain of *E. coli* (K-12) elicited a less pronounced flagellin response than did a pathogenic strain of *S*. Typhimurium [89]. Divergence in the protofilament number in *C. jejuni* compared to *S*. Typhimurium serves to evade the TLR5 recognition, due to substantially different packing of the D1 domains [90].

Immune activation in plant hosts varies depending on variation in the flg22 sequence of not only phytopathogenic bacteria [76,77,88,91] but also human pathogenic bacteria. Variation in five amino acids of the flg22 peptide derived from certain *S*. Senftenberg isolates induces a reduced pattern-triggered immunity (PTI) in plants in comparison to that from *S*. Typhimurium [92]. Likewise, plant growth-promoting rhizobacteria (PGPRs), such as *Sinorhizobium meliloti* and *Agrobacterium tumefaciens* have divergent flg22 epitopes that do not elicit any responses [75]. There is also evidence to suggest that the PGPR *Burkholderia phytofirmans* has evolved to evade the grapevine immune recognition system via FLS2 altogether [93].

As an alternative strategy, some bacteria evade immune recognition of flagellin independently of the flg22 peptide (Fig. 3). Phytopathogenic bacteria secrete effector proteins that specifically target FLS2 counteracting detection in plants. While some effectors rapidly act on FLS2 to shut down the PTI response [94,95], others suppress FLS2 accumulation and subsequent signalling cascades [96]. FLS2-induced stomatal closure is a characterised response to prevent pathogens from entering internal plant tissue [97], dependent on the action of oxylipins, rather than the phytohormone abscisic acid (ABA) [98]. Some isolates of *P. syringae* can prevent stomatal closure via secretion of a phytotoxin (coronatine) [99]. Intriguingly, *S*. Typhimurium can also delay stomatal closure, although to a lesser extent, via potential effector proteins other than coronatine, which may target the oxylipin pathway. EHEC O157:H7, which shares the same flg22 sequence as *S*. Typhimurium, cannot prevent stomatal closure, suggesting active manipulation of PTI by *Salmonella* [100]. *P. aeruginosa*, an opportunistic pathogen of plants and animals, produces an alkaline protease (AprA) to specifically degrade flagellin monomers. This strategy effectively evades the immune recognition in both kingdoms by degrading the natural ligand of TLR5 and FLS2 [101].

### Post-translational modification

An alternative mechanism of evading recognition may come from modification of flagellin, reported for a number of animal and phytopathogenic bacteria [102,103]. Glycosylation enhances the structural stability of flagellin, preventing exposure of the flg22 region to FLS2 recognition, thereby evading the plant immune response [104]. There are multiple examples of *O*-glycosylation, well characterised for the flagellins of *C. jejuni* and *Campylobacter coli* [105]. Other examples include *P. aeruginosa* [106] and *Shewanella oneidensis* [107]. However, *C. jejuni* flagellar glycosylation was not involved in evasion of TLR5 recognition [108]. Perhaps it relates more to virulence and adherence, rather than filament stability [109–111]. Interestingly, *S*. Typhimurium flagellin is methylated at multiple lysine residues, yet this has no known impact on flagellar function [112]. The role of flagella post-translational modification in immune recognition clearly needs further investigation.

## Concluding Remarks

Flagella enable pathogens to exploit or capitalise on various niches associated with the host. Although they display a range of functions, these are intrinsically linked to host colonisation and their own biophysical properties. Flagella are therefore not a virulence factor per se, but rather an early stage colonisation factor. They facilitate individual, pioneering cells to access, bind and invade new plant and animal tissues, and if successful in avoiding host recognition and clearance, to establish new colonies. More work is needed to understand how bacteria progress from flagella expression to flagella disassembly, both in the context of expression of more specialised colonisation factors that target specific ligands, and in immune recognition. The location of known ligands and the differences between decoys and membrane bound receptors also need to be addressed. Undoubtedly, future research will uncover further surprises for “twist and stick” or “dodge” flagella.
